# Supplementary material for: Causal interplay between lactose intolerance and gut microbiota: a combined bidirectional Mendelian randomization and in vivo validation study
Source: Front Nutr. 2026 Jun 1;13:1803337. doi: 10.3389/fnut.2026.1803337 (PMC13265576; doi:10.3389/fnut.2026.1803337)
Supplement: Supplementary file 1 [file Data_Sheet_1.zip › supplementary materials/Forward/leave one out cross validation/ebi-a-GCST90027505.finngen_R12_E4_LACTONAS.pdf]

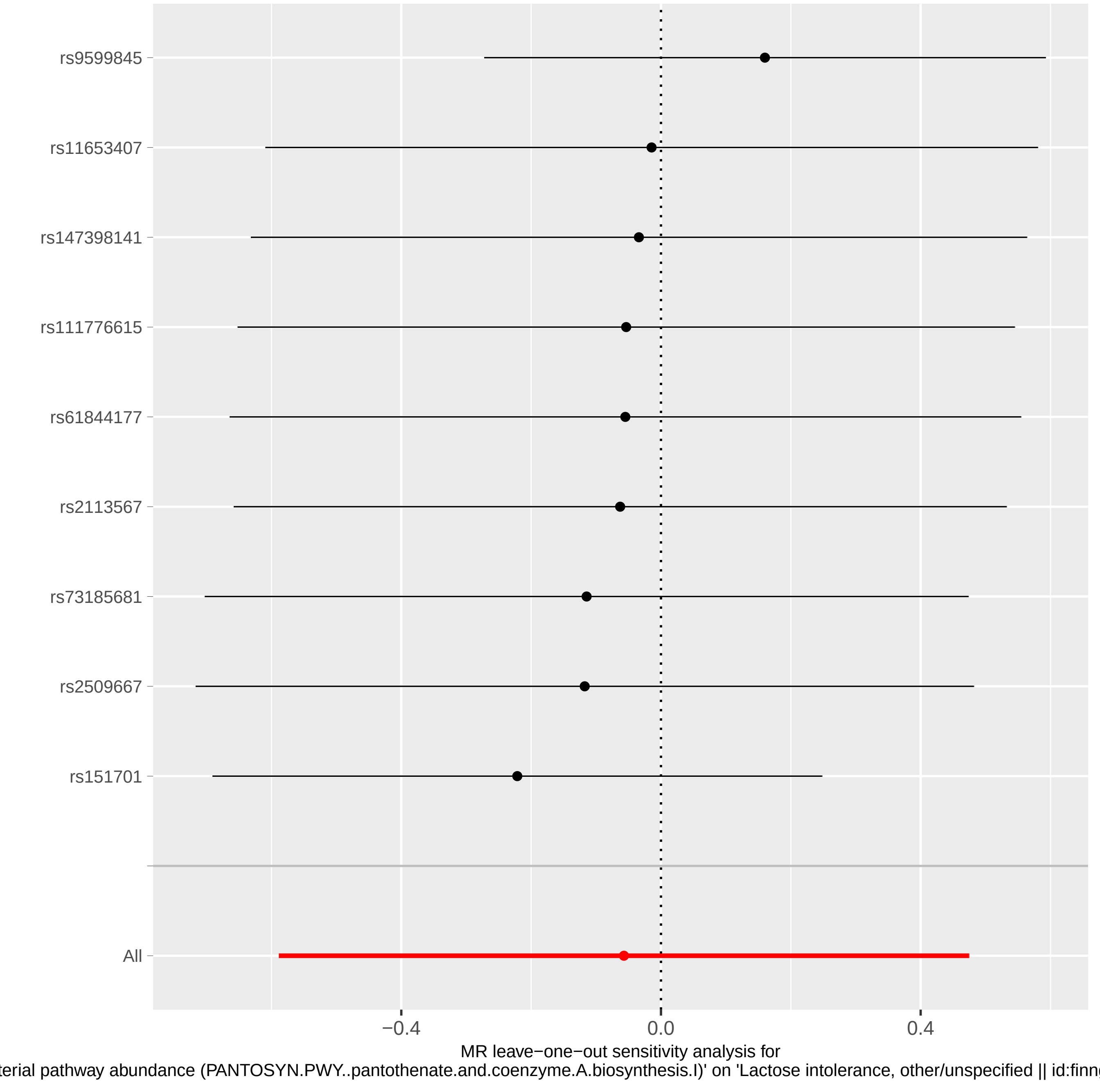
rs9599845
rs11653407
rs147398141
rs111776615
rs61844177
rs2113567
rs73185681
rs2509667
rs151701
All
−0.4
0.0
0.4
MR leave−one−out sensitivity analysis for
terial pathway abundance (PANTOSYN.PWY..pantothenate.and.coenzyme.A.biosynthesis.I)' on 'Lactose intolerance, other/unspecified || id:finn
